# Ghrelin receptor controls obesity by fat burning

**DOI:** 10.18632/oncotarget.3668

**Published:** 2015-03-27

**Authors:** Yuxiang Sun

**Affiliations:** USDA/ARS Children's Nutrition Research Center, Huffington Center on Aging, Baylor College of Medicine, Houston, TX, USA

Obesity has reached epidemic proportions in all age groups, and is more pronounced in the elderly. Obesity is the most prominent risk factor for insulin resistance, type 2 diabetes, and cardiovascular disease. Obesity is centered in adipose tissues, which are designated as either energy-storing white adipose tissue (WAT) or energy-burning brown adipose tissue (BAT). Emerging evidences show that non-shivering thermogenesis plays a crucial role in regulation of energy homeostasis in both rodents and humans [1-3]. Thermogenesis positively correlates with energy expenditure, but negatively correlates with body fat. Dysfunction of thermogenesis decreases energy expenditure, promoting obesity. Aging is associated with severe thermogenic impairment. PET/CT scans show that BAT declines 95% in mass and 75% in activity in old men compared to young men [2]. Beside the classical brown adipocytes present in BAT, brown-adipocyte-like “beige” cells residing in WAT also have thermogenic properties [3]. Aging is associated with programmed loss of “beige” cells [4]. Animal studies reveal that thermogenic activation in brown and beige adipocytes protects against obesity. These discoveries suggest that pharmacologically activating thermogenesis might be a powerful means of combating age-associated obesity. However, currently little is known about thermogenic regulation during aging.

WAT and BAT have distinct characteristics and functions. WAT stores energy as triglycerides in large unilocular lipid droplets; WAT supplies energy to the body via lipolysis. In contrast, BAT contains adipocytes with multi-locular lipid droplets and high-density mitochondria; BAT consumes energy to produce heat [1]. Upon cold or diet challenge, nerve endings of the sympathetic nervous system (SNS) release norepinephrine (NE) to activate β3- adrenergic receptors (β3-AR) in brown adipocytes. The β3-AR signaling activates protein kinase A (PKA), which then phosphorylates hormone-sensitive lipase (HSL) to increase lipolysis of lipid droplets; this results in release of glycerol and free fatty acids (FFA). Uncoupling protein 1 (UCP1) is the hallmark regulator of thermogenesis. FFA activates UCP1 in mitochondria to pump protons into the mitochondrial matrix to dissipate heat [1].

Ghrelin is the only known circulating orexigenic hormone; it promotes meal initiation, adiposity, and insulin resistance. The dogmatic view is that ghrelin regulates metabolism primarily by affecting food intake. Growth Hormone Secretagogue Receptor (GHS-R) is the receptor for ghrelin. We and others have shown that GHS-R is highly expressed in hypothalamus, but its expression in peripheral tissues is very low in young mice. Intriguingly, circulating ghrelin and GHS-R expression in the brain and adipose tissues increase during aging [4, 5]. We have reported in *Aging Cell* that old *Ghsr*-null mice are lean and insulin-sensitive, exhibiting a healthier metabolic profile similar to that of young animals [6]. Metabolic profiling analysis indicates that the reduced adiposity observed in old *Ghsr*-null mice is due to increased energy expenditure, but not due to reduced food intake or increased physical activity [6]. Old *Ghsr*-null mice have higher core body temperature and show better resistance to cold, consistent with increased thermogenic activity in BAT [6]. Our findings indicate for the first time that ghrelin signaling plays an important role in thermogenesis during aging; GHS-R regulates energy homeostasis by burning fat to generate heat, not by reducing long term energy intake. This challenges the dogmatic view of ghrelin's regulation of lipid metabolism via its orexigenic property, and reveals an exciting possibility that suppressing ghrelin signaling may protect against age-associated obesity by enhancing thermogenesis.

**Figure 1:**
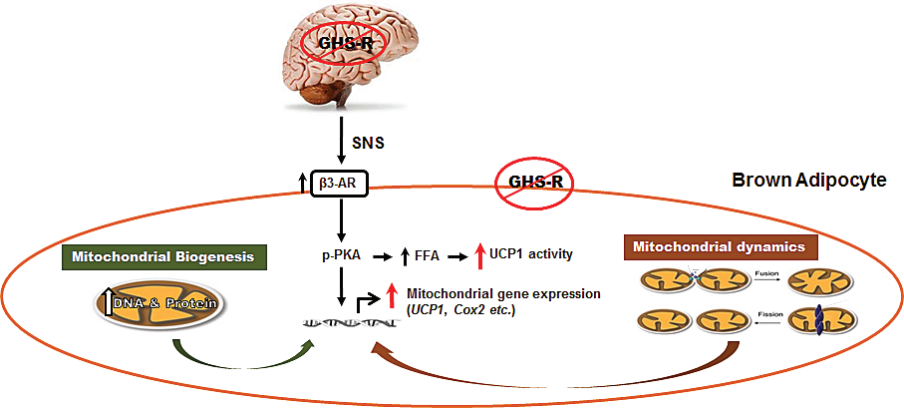
Schematic diagram of GHS-R mediated thermogenic regulation in brown adipocytes GHS-R may regulate thermogenesis in BAT via the following signaling pathways: 1) Ablation of GHS-R stimulates SNS-mediated NE release, which in turn induces β3-AR expression, subsequently activating thermogenic signaling cascades in BAT. This involves activation of thermogenic signaling pathway PKA-CREB-UCP1 and lipolytic pathway PKA-HSL-UCP1. 2) Ablation of GHS-R enhances increases DNA and protein synthesis of mitochondria, thus increasing mitochondrial biogenesis. 3) Ablation of GHS-R augments mitochondrial dynamics, enhancing both mitochondrial fission and fusion; this restores mitochondrial architecture and improves mitochondrial homeostasis. Collectively, GHS-R ablation increases thermogenesis in BAT by activating thermogenic signaling, increasing mitochondrial biogenesis, and enhancing mitochondrial dynamics. (*Modified from Aging 6:1019*).

Mitochondria are the powerhouses of cells; mitochondrial energetics is determined by biogenesis and dynamics (consisting of fission and fusion). Dysfunctions of mitochondrial energetics is linked to dysfunction of aging neuronal and muscular systems [7], but the roles of mitochondria in thermogenic regulation during aging are not clear. Our recent studies recently published in *Aging* have revealed that GHS-R ablation activates thermogenic signaling in BAT by enhancing mitochondrial biogenesis, and improving both mitochondrial fission and fusion in brown fat (Figure 1) [8]. In addition, we have detected increased norepinephrine in the circulation and higher β3-AR expression in BAT of old *Ghsr*-null mice; and that GHS-R knockdown in brown adipocytes directly stimulates thermogenic activity. These results suggest that GHS-R may, via both central and peripheral mechanisms, regulate the thermogenic signaling cascade in BAT.

Collectively, our studies demonstrate that GHS-R is a novel thermogenic regulator which plays an important role in lipid metabolism during aging. Increased circulating ghrelin and up-regulation of GHS-R during aging may contribute to age-associated thermogenic impairment, thus increasing incidences of obesity in aging. GHS-R ablation prevents the age-associated decline of thermogenesis, thus reducing obesity and improving insulin sensitivity. The healthy phenotype of *Ghsr*-null mice remarkably resembles the effects of the most recognized anti-aging intervention, calorie restriction. Collectively, these “proofof- concept” studies suggest that GHS-R antagonists may serve as unique anti-obesity agents, combating obesity by enhancing thermogenesis and shifting metabolic state from obesogenic to thermogenic. Further studies are needed to identify the sites of action and cellular/molecular mechanisms, and to determine the role of GHS-R in thermogenic dysfunction in humans.
